# Breaking the cycle: should we target inflammation, fibrosis, or both?

**DOI:** 10.3389/fimmu.2025.1569501

**Published:** 2025-09-08

**Authors:** Sarah K. Baxter, Ricardo A. Irizarry-Caro, Jason A. Vander Heiden, Joseph R. Arron

**Affiliations:** ^1^ Sonoma Biotherapeutics, South San Francisco, CA, United States; ^2^ Sonoma Biotherapeutics, Seattle, WA, United States; ^3^ Genentech, Inc., South San Francisco, CA, United States

**Keywords:** interstitial lung disease, fibrosis, inflammation, IL6, TGFβ, B cells

## Abstract

Left unchecked, many chronic inflammatory and autoimmune diseases lead to fibrosis, which can ultimately irreversibly compromise tissue and organ function. A key question for therapeutic discovery and development is whether it is preferable to target inflammation, fibrosis, or both; and in which tissues, organs, diseases, or subsets of patients is a particular therapeutic strategy most relevant? In recent years, clinical and translational studies of human interstitial lung disease tissue and targeted molecular and cellular therapies have yielded mechanistic insights into the interplay between unchecked inflammation and pathological fibrogenesis. Molecular and proteomic technologies have implicated aspects of both innate and adaptive immunity in fibrogenesis, e.g., the presence of a stereotypical population of fibrosis-associated macrophages, recruitment of immune cells by inflammatory fibroblasts, and lymphoid aggregates with B cells producing tissue-specific autoantibodies. In this Perspective, we will consider indications that present with inflammation and/or fibrosis in lung tissue, including systemic sclerosis (SSc), idiopathic pulmonary fibrosis (IPF), and rheumatoid arthritis (RA), in the context of clinical and translational data from molecular interventions targeting cytokine pathways and B lymphocytes. The effects of these interventions on clinical, functional, cellular, and molecular outcomes have started to untangle the mechanistic relationships between inflammation and fibrosis in human diseases, and may illuminate a path toward improved strategies to restore tissue homeostasis and preserve or improve functional outcomes in the future. However, substantially more granular clinical outcomes, biomarker data, and assay standardization across interventions and diagnoses are needed to effectively link therapeutic targets, disease pathophysiology, and clinical benefit.

## Introduction

Despite numerous therapies targeting inflammation, fibrosis remains an intransigent manifestation of many inflammatory diseases. It is unclear whether fibrosis is a distinct pathologic process alongside dysregulated immune responses, a downstream consequence of unchecked inflammation, or both. This distinction is critical for guiding treatment strategies. If fibrosis is an independent process distinct from a dysregulated immune response, then therapies should directly target fibrotic mechanisms. Conversely, if fibrosis arises as a sequela of chronic inflammation and tissue damage, then targeting inflammation may inherently mitigate fibrosis. This interplay is particularly evident in interstitial lung disease (ILD). ILD emerges as a shared manifestation across diseases of autoimmunity, environmental exposures, and conditions driven by aberrant tissue repair pathways (1, 2). While various ILDs may have different initiating factors, many pathobiological features including inflammatory processes and tissue fibrosis are common in established disease across diagnoses.

## Clinical aspects of inflammatory and fibrotic ILDs

Interstitial lung diseases encompass a spectrum of conditions with diverse etiologies, clinical presentations, and histopathologic patterns. ILDs associated with connective tissue diseases (CTD-ILD) and idiopathic pulmonary fibrosis (IPF) share overlapping features while presenting unique challenges in diagnosis and management. IPF is an archetypal fibrotic ILD. It is rapidly progressive and often fatal, primarily affecting adults over the age of 50, with a median survival after diagnosis of 3–5 years (3). Despite the term “idiopathic,” emerging genetic and mechanistic data strongly suggest that insufficient alveolar epithelial regenerative capacity in the face of chronic epithelial damage is the initiating etiological factor in IPF (4, 5). While the proximal cause of fibrosis in IPF lies within injury and repair pathways, there is clear evidence for inflammation in established disease. Treatment options aim to slow progression, manage symptoms, and improve quality of life, but no interventions to date have produced meaningful reversal of the progressive loss of lung function. Current approved IPF treatments include pirfenidone and nintedanib, which show modest efficacy in slowing lung function decline and prolonging survival in some patients (6). Both are associated with significant tolerability issues, and many patients proceed to lung transplantation. Neither drug has a clearly defined molecular or cellular mechanism of action. Pirfenidone is alleged to have both anti-inflammatory and anti-fibrotic effects but neither has been clearly documented in humans; nintedanib partially inhibits dozens of kinases at *in vivo* exposures but which of those is responsible for its therapeutic benefit remains unclear (7, 8). Broad-spectrum anti-inflammatories, including steroids and cyclophosphamide, were previously used but are now contraindicated due to increased mortality (9). This lack of responsiveness to nonspecific anti-inflammatory treatment highlights a divide between IPF and the Connective Tissue Disease-associated ILDs (CTD-ILD), such as systemic sclerosis-ILD (SSc-ILD) and rheumatoid arthritis-associated ILD (RA-ILD).

Whereas genetic associations with IPF generally implicate lung epithelium, genetic associations with SSc generally implicate immunity (10, 11). SSc is hypothesized to originate as small vessel autoimmune vasculitis, which can progress to progressive fibrosis affecting the skin, lungs, heart, kidneys, and/or gastrointestinal tract (12). Lung involvement, and in particular ILD, occurs in up to 70% of patients with diffuse SSc and is the leading cause of mortality (13). Similar to IPF, the interplay of inflammation, tissue injury, and tissue repair confound our understanding of the pathogenesis of SSc-ILD. SSc patients often demonstrate chronic activation of both the innate and adaptive immune systems (14). Autoantibodies, such as anti-topoisomerase I and anti-RNA polymerase III, are diagnostic markers and may contribute to immune activation and tissue injury (15, 16). Persistent immune activation, in addition to perpetuating a cycle of inflammation and fibrosis, also disrupts normal tissue repair pathways, and targeting inflammation remains a cornerstone of SSc therapy (17). However, the limited efficacy of current treatments underscores the need for further research into fibroblast biology and dysregulated tissue repair mechanisms.

Rheumatoid arthritis-associated ILD (RA-ILD) is an increasingly recognized cause of morbidity and mortality in RA patients. Occurring in approximately 10% of RA patients, RA-ILD predominantly affects older males and those with a history of smoking. RA-ILD significantly impacts morbidity and mortality in RA patients, with a median survival of 3–8 years following diagnosis (18). Similar to IPF, imaging reveals fibrosis, honeycombing, and ground-glass opacities in affected individuals. The pathogenesis of RA-ILD is unclear, but, like other CTD-ILDs, likely involves a complex interplay of chronic inflammation, tissue injury, and aberrant repair. Autoantibodies such as rheumatoid factor (RF) and anti-citrullinated protein antibodies (ACPA) may contribute to lung injury (19–21). Treatment strategies primarily are aimed at inflammatory pathways, though efficacy is limited, and many therapies that can effectively treat arthritis, such as TNF-α inhibitors, do not appear efficacious in treating ILD (22, 23).

ILD can present in patients with other rheumatic diseases as well as in allogeneic hematopoietic stem cell transplant recipients. Given the wide and overlapping clinical subtypes of ILD, understanding the histopathologic pattern is key to diagnosis, prognosis, and treatment (24). Two patterns most associated with fibrotic potential include usual interstitial pneumonia (UIP) and non-specific interstitial pneumonia (NSIP). UIP, the most common pattern in IPF, carries a poor prognosis, with a median survival from time of diagnosis of 3–5 years, and limited responsiveness to immunosuppression (24). NSIP is more common in CTD-ILDs and has a more favorable prognosis; ILD with a NSIP pattern can respond to immunosuppressive treatments (25). However, there is considerable pathological heterogeneity across ILD diagnosis, and CTD-ILD patients with a UIP pattern tend to have similarly poor prognosis to patients with IPF (26). Taken together, these observations suggest that understanding mechanistic heterogeneity and prognosis may be more useful than clinical classifications for guiding treatment strategies. Clinical trials for ILD face numerous challenges due to the complexity, heterogeneity, and progressive nature of these diseases. Traditional trial endpoints such as forced vital capacity (FVC) and mortality require long trial durations, and may not fully capture disease burden or patient-centered outcomes (27). Given these challenges, it will be critical for the field to identify biomarkers and patient-centered endpoints that can capture disease activity and assess meaningful outcomes in a shorter trial duration.

## Cellular and molecular correlates of inflammation-associated fibrosis

**Figure 1:**
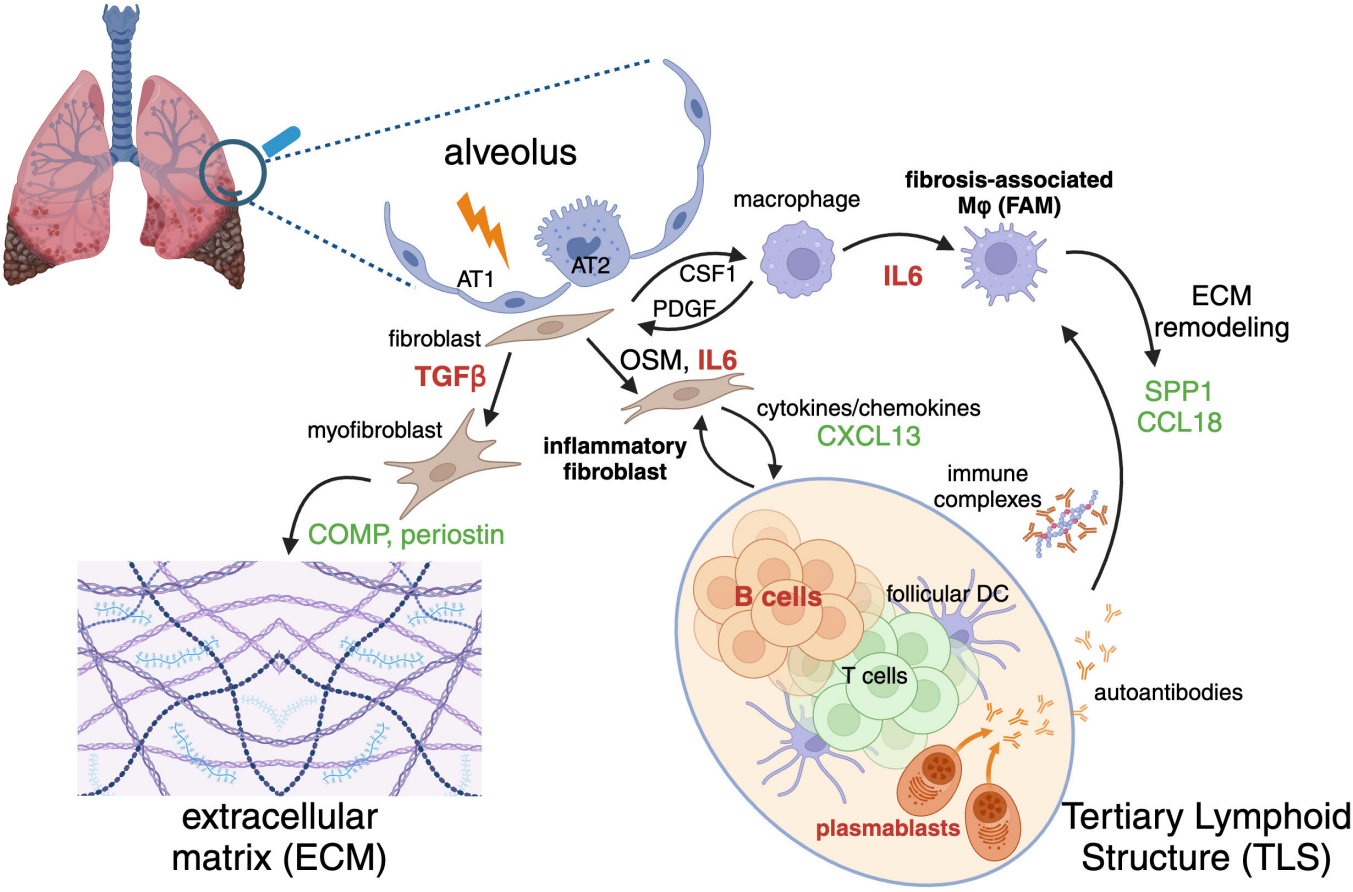
Inflammatory and fibrotic cycle of cells, structures, molecular mediators, therapeutic targets, and biomarkers implicated in fibrotic interstitial lung diseases (ILD). Injury or damage to alveolar epithelial cells (AT1, type 1 alveolar epithelial cell; AT2, type 2 alveolar epithelial cell) initiates activation of fibroblasts and macrophages. Fibroblasts can differentiate into extracellular matrix (ECM)-producing myofibroblasts or cytokine-producing inflammatory fibroblasts. Macrophages differentiate into ECM-remodeling fibrosis-associated macrophages (FAM). Adaptive immune cells including B and T lymphocytes constitute tertiary lymphoid structures (TLS). A variety of factors mediate cross-talk between these inflammatory and fibrosis-perpetuating cells. Selected targets of therapeutic interest discussed in the text are indicated in red (TGF-β, IL-6, and B cells) and biomarkers related to their activity are indicated in green. Figure created with Biorender.

Fibrotic ILDs involve a complex interplay of cellular interactions within lung tissue, characterized by unchecked inflammation, extracellular matrix (ECM) deposition, and progressive tissue remodeling. This pathological transformation is driven by a microenvironment of pro-inflammatory and pro-fibrotic cell populations, including macrophages, lymphocytes, and fibroblasts, which form complex cell-cell communication networks that perpetuate disease (**Figure 1**).

While precipitating fibrosis-driving micro-injuries vary by indication, common damage response signals lead to activation of resident macrophages and fibroblasts, which then enter pro-inflammatory and pro-fibrotic states. These activated cells secrete a host of cytokines and chemokines, such as TNFα, IL1β, CSF1, and IL6, that facilitate the recruitment and subsequent infiltration of lymphocytes, monocytes, and neutrophils to sites of tissue injury (28–32). This influx of immune cells further amplifies the inflammatory milieu, perpetuating a cycle of chronic inflammation and fibrosis. Macrophages and fibroblasts participate in a CSF1-PDGF positive feedback loop, wherein CSF1 produced by fibroblasts supports the maintenance and expansion of macrophages. In response, macrophages secrete PDGF, a potent mitogen that drives fibroblast proliferation and differentiation into myofibroblasts. This interaction mirrors the *in vivo* aberrant wound repair processes observed in fibrosis, where a runaway injury response leads to nonresolving ECM deposition and tissue remodeling (33, 34).

Beyond this minimal cell communication model, the *in vivo* cross-talk between macrophages and fibroblasts encompasses several other pro-fibrotic mediators. Macrophage-derived OSM and fibroblast derived TGFβ and IL6 play pivotal roles in promoting fibroblast activation and lymphocyte differentiation, while macrophage expression of MMPs and TIMPs regulates ECM turnover and remodeling. Additionally, chemokines such as CCL2 and CCL11 recruit various immune cells, further contributing to the fibrotic cascade (35–37). Fibrosis-associated macrophages (FAMs) and myofibroblasts with unique phenotypic hallmarks are commonly observed across a spectrum of fibrotic disorders, including IPF, SSc-ILD, COVID-19, non-alcoholic fatty liver disease, pancreatic ductal adenocarcinoma, and heart disease (38–40). Despite tissue-specific differences in gene expression profiles, these cells exhibit a core set of features. FAMs are characterized by the expression of genes involved in lipid metabolism and ECM remodeling, such as *APOC1, APOE, CHIT1, LPL, LIPA, LGMN, PLA2G7, MMP9*, and *MMP7*. Additionally, genes associated with wound repair and injury response, including *TREM2, SPP1, CCL18*, and *GPNMB*, are enriched in these macrophages (38, 39, 41, 42). Disease-associated fibroblasts can be broadly categorized into inflammatory fibroblasts and myofibroblasts, demonstrating significant subtype heterogeneity. At a high level, inflammatory fibroblasts can be defined by a tissue-context-dependent cytokine and chemokine secretion gene expression program, which may include *IL6, IL33, CCL1, CXCL2, CXCL3, CXCL6, CXCL8, CXCL12, CXCL13*, and *CXCL14*. In contrast, myofibroblasts are distinguished by canonical ECM deposition and contractile programs, including *ELN, POSTN, COMP, TNC, COL1A1, COL3A1*, and secrete pro-fibrotic factors such as TGFβ3, which further perpetuate the fibrotic cycle (36, 43–48).

A notable feature of many ILDs is the infiltration of adaptive immune cells and the formation of tertiary lymphoid structures (TLS), characterized by increased expression of the activation marker CD40 within aggregates of B cells surrounded by CD8^+^ and CD4^+^ T cells. This adaptive immune response is closely linked to disease progression and severity (49), although it remains unclear whether it is a driver or a consequence of advanced tissue destruction. CXCL13, a major B cell chemoattractant, is produced by follicular dendritic cells, reticular fibroblasts, and T cells and its induction by activated fibroblast-derived TGFβ has been observed in lung cancer (50), suggesting a similar mechanism may drive TLS formation in fibrotic lungs.

Understanding these intricate networks of cellular communication in fibrotic lung tissue is crucial for developing therapeutic strategies that target these interactions. By intervening in these pathways, we may be able to modulate the inflammatory and fibrotic responses, potentially halting or reversing the progression of these debilitating diseases.

## Therapeutic targeting of cytokines and leukocytes in fibrotic diseases

The interplay between inflammation and fibrosis is central to the pathogenesis of chronic fibrotic diseases. Understanding the mechanisms underlying this relationship offers opportunities for developing therapeutic interventions that target both processes. We have explored cytokines such as IL6 and TGFβ as therapeutic targets. Additionally, interventions aimed at eliminating B cells, such as anti-CD20 therapy (e.g., rituximab) or cellular therapies like CD19-directed CAR-T cells, have been investigated in fibrotic disorders.

IL6 is a multifunctional cytokine that bridges innate and adaptive immunity. In SSc and IPF, elevated IL6 levels correlate with disease severity and progression (51–53). Mechanistically, IL6 promotes fibroblast activation and ECM deposition through the JAK/STAT3 pathway. Additionally, it drives T-helper 17 (Th17) responses (54) and fibrotic macrophage polarization (35), exacerbating and perpetuating a maladaptive cycle of inflammation and fibrosis. IL6 blockade using monoclonal antibodies, such as tocilizumab (anti-IL6Rα), has shown promise in attenuating fibrosis and inflammation in both preclinical and clinical studies. By inhibiting IL6, inflammation is reduced, fibrotic programs are impaired, fibroblast activation is suppressed, and ECM deposition is limited. In two placebo-controlled trials, tocilizumab demonstrated benefits in SSc-ILD by preserving lung function; however, it did not meaningfully affect skin fibrosis (55, 56).

Transforming Growth Factor-β (TGFβ) is considered the archetypal pro-fibrotic factor, with well-established roles in fibrosis across multiple organs (32). TGFβ drives fibroblast-to-myofibroblast differentiation and collagen production, inhibits ECM degradation, and modulates immune cell functions to sustain a fibrotic microenvironment. However, the pleiotropic nature of TGFβ demands careful therapeutic balancing to avoid disrupting tissue integrity and immune homeostasis. TGFβ comprises three highly homologous cytokines (TGFβ1, TGFβ2, and TGFβ3) that, in their active forms, activate similar cellular signals through TGFβ receptors (57–60). These isoforms are differentially expressed across cell and tissue types in a latent state and are activated via distinct mechanisms, which together tightly regulate their diverse effects *in vivo*. In SSc, TGFβ blockade with fresolimumab, a pan-TGFβ inhibitor, yielded mixed results, showing modest anti-fibrotic benefits in some studies but failing to meet primary endpoints in others, along with raising safety concerns (61). In IPF, TGFβ inhibitors, including fresolimumab and the peptide-based HTPEP-001 demonstrated preclinical promise, but clinical trials have produced inconsistent outcomes (62). We have recently shown that isoform-selective inhibition of TGFβ3 may attenuate fibrosis without inducing excessive inflammation (63), and that isoform-selective TGFβ3 targeting is safe and well-tolerated in preclinical toxicology studies [PMID:40317127], representing a refined therapeutic approach that is currently under clinical investigation in SSc.

B cells contribute to inflammation and fibrosis through antibody production, antigen presentation, and cytokine secretion. In diseases such as SSc and IPF, B cells are enriched in fibrotic lesions, where they may promote fibroblast activation and ECM remodeling. Rituximab, a monoclonal antibody targeting CD20, has shown promise in improving skin thickening and stabilizing lung function in diffuse cutaneous SSc (64), as well as potential benefits in treating SSc-associated pulmonary arterial hypertension (65). In IPF, however, CD20-targeting therapies have demonstrated limited efficacy, with emerging evidence suggesting a potential role of CD20-negative B lineage cells such as plasma cells in driving fibrosis. Deeper B cell depletion strategies, such as CD19-targeted CAR-T cell therapy, are currently under investigation, with limited anecdotal data indicating potential efficacy in SSc (66). It remains unclear whether the therapeutic effects of B cell targeting in fibrotic disorders are linked to the presence of tertiary lymphoid structures (TLS). In a subset of patients with rheumatoid arthritis (RA), the presence of TLS in the synovium has been associated with greater clinical benefits from both rituximab and tocilizumab (67). Whether similar associations exist in fibrotic ILDs represents an intriguing question for future research. While the presence of TLS correlates with ILD severity and prognosis, it remains unclear whether they are a therapeutically actionable driver of ILD progression or a secondary consequence of extensive damage to a barrier tissue.

## Biomarkers and translational insights from clinical studies

Biomarkers in clinical studies of patients with fibrotic disorders have yielded important mechanistic insights into the roles of inflammatory and fibrotic molecular and cellular processes and their relationships to clinical manifestations and response to treatment. Peripheral blood is readily accessible and amenable to repeat sampling over time. As fibrotic ILDs are generally patchy and diffuse throughout the lung parenchyma, an ideal blood biomarker would reflect molecular or cellular processes relevant to disease pathogenesis and “spill over” into circulation sufficiently such that its levels can integrate the aggregate burden of those processes across total lung tissue (68). In the context of a therapeutic intervention, a given biomarker may be predictive, prognostic, and/or pharmacodynamic; i.e., it may identify patients more or less likely to exhibit clinical response to therapy, it may identify patients at greater or lesser risk of significant disease progression, and/or it may change in response to the administration of a therapeutic agent (69).

Many transcripts that characterize fibrosis-associated macrophages encode secreted soluble proteins that can be readily detected in peripheral blood, e.g., SPP1, GPNMB, and CCL18 (70–74). In the case of SPP1 and CCL18, elevated blood levels are associated with ILD severity. ILD patients with higher levels of blood CCL18 at a given time have an increased risk of disease progression, hence CCL18 is prognostic (69, 75). In SSc-ILD patients treated with tocilizumab (anti-IL6Rɑ), CCL18 levels rapidly declined, exhibiting a pharmacodynamic response to IL6 inhibition, and lung function decline was significantly attenuated in tocilizumab-treated as compared to placebo-treated patients despite a lack of significant benefit on skin fibrosis outcomes (76, 77). These observations, taken together, suggest that IL6 can promote a FAM phenotype, which is associated with ILD severity, and that a mechanism of clinical efficacy of IL6 inhibition in ILD may be by altering macrophage biology.

Activated fibroblasts and myofibroblasts secrete large quantities of proteins to constitute the ECM, but many of these proteins are highly matrix-associated, and their circulating levels may not always reflect tissue activity. However, periostin and COMP, TGFβ-inducible matricellular proteins highly expressed in myofibroblasts, “spill over” sufficiently that they are readily detectable in peripheral blood and are significantly elevated in patients with fibrotic disorders (69). In SSc, whereas peripheral CCL18 is primarily associated with ILD, blood periostin levels are highly correlated with the extent of skin fibrosis but are not related to the presence nor severity of ILD (78). While tocilizumab treatment exerted significant pharmacological effects on blood CCL18 and clinical benefit on lung function, it did not affect blood periostin levels nor the extent of clinical skin fibrosis (76). Taken together, these observations suggest that fibrotic disease progression in SSc may be driven by distinct mechanisms in lung and skin, where IL6 and fibrosis-associated macrophages are more closely associated with lung function while other targets such as TGFβ and myofibroblasts may be more closely associated with skin fibrosis. Conversely, in SSc patients treated with the TGFβ inhibitor fresolimumab, reductions in skin gene expression of COMP but not the macrophage marker CD163 suggest that TGFβ inhibition affected fibroblast but not macrophage biology in the skin (79). Future studies comprehensively assessing a full array of these biomarkers locally and systemically in the context of specific molecular interventions have the potential to yield deeper insights into cross-talk between pathways and cells across diseases and tissues.

TLS in lung tissue are associated with disease activity in fibrotic ILDs. In IPF, lymphoid aggregates are observed in regions of more advanced disease with significant breakdown of alveolar architecture and dense extracellular matrix deposition (49, 68). CXCL13 recruits lymphocytes via CXCR5 to TLS. Elevated blood CXCL13 levels are associated with increased lymphoid aggregates in multiple inflammatory diseases associated with antigen-dependent autoimmunity including RA, SLE, multiple sclerosis, myasthenia gravis, and Sjögren’s Syndrome (80). In IPF, CXCL13 was prognostic for loss of lung function and mortality (68, 81), suggesting that increased aggregate burden of lymphoid aggregates across lung tissue was associated with disease severity. B cell depletion with rituximab (anti-CD20) has not consistently demonstrated benefit in patients with fibrotic ILD in controlled studies (82–84), however substantial clinical activity in SSc was anecdotally reported with CD19-directed CAR-T or anti-CD3/CD19 T cell engager treatment, albeit in open-label investigations of small numbers of patients with limited follow-up and potentially concerning toxicity (85–87). If greater benefit for CD19-directed CAR-T treatment than anti-CD20 antibodies can be substantiated in larger, well-controlled trials, it is possible that the greater degree of tissue B cell depletion and/or targeting a broader range of B cells including plasmablasts may explain these differences. Interestingly, we found that a tissue gene expression signature corresponding to plasma cells in skin and lung was associated with prognosis in SSc and demonstrated a pharmacodynamic response to tocilizumab treatment (88), suggesting that IL6 modulation of adaptive immunity may also contribute to SSc pathogenesis.

## Perspective

Inflammation and fibrosis require more precise definitions in molecular, cellular, and tissue terms. In the case of ILDs, while there are diverse underlying initiators of disease, it may be more clinically useful to redefine them in terms of targetable biology and prognosis using biomarkers. While many interventions have been explored clinically, a more comprehensive understanding of the interplay between their mechanisms of action is needed; biomarkers may provide some insight but have not been systematically investigated across diagnoses and interventions. Ideally, biomarkers related to specific disease processes including but not limited to FAMs, inflammatory fibroblasts, and TLS could be assessed for prognostic, predictive, and pharmacodynamic effects in basket-style studies across multiple clinical diagnoses and therapeutic mechanisms of action. These results could deconvolve which processes are affected by a given therapy and which processes may underlie limited efficacy on various outcome measures. Although significant challenges exist in standardizing assay platforms and reconciling data across a broad range of clinical studies, such efforts may significantly help to advance our understanding. New therapies should aim to restore homeostasis by disrupting the maladaptive cycle of inflammation and fibrosis and create a permissive environment for functional tissue regeneration. Specific targeting of a single molecular mediator may be insufficient; emerging therapies that target multiple pathways or cellular therapies with pleiotropic effects and the ability to respond to evolving tissue milieus may offer the potential to tame pathogenic processes and promote homeostasis. In that regard, the lack of a single identifiable molecular target may explain the therapeutic efficacy, albeit limited, of pirfenidone and nintedanib. However, future therapies will need to have greater potency and be sufficiently safe, well tolerated, and convenient to sustain long term disease modification.

## Data Availability

The original contributions presented in the study are included in the article/supplementary material. Further inquiries can be directed to the corresponding author.
